# Inflammation

**Published:** 1845-01

**Authors:** 


THE PHYSIOLOGY OF INFLAMMATION AND THE HEALING PROCESS. By *Benjamin Travers*, F.R.S. 1844. 8vo, pp. 226.

TREATISE ON INFLAMMATION AS A PROCESS OF ANORMAL NUTRITION. By *John Hughes Bennett*, M.D., F.R.S.E. 1844. Octavo, pp. 80.

FROM the period of the researches of Hunter to the present day the subject of Inflammation has proved one of surpassing and abiding interest. That this should be the case can excite no surprise; for, while a correct theory of its production may amend or confirm many of the views of the physiologist, and clear up some of the numerous obscurities which beset the path of the pathological inquirer, the mere practical man, in deriving hence a knowledge of the true principles upon which its treatment should be based, becomes the possessor of by far the most powerful instrument for the relief of disease. It is true that the crude and exaggerated notions, according to which inflammation forms the basis and point of departure of all diseases, have done infinite mischief, not only by the ultra-depletory practices they encouraged during the day of their prevalence, but also by reason of opposite errors which have resulted from the re-action consequent upon their explosion; and yet the justice of Mr. Lawrence's statement, that few indeed are the diseases in which the inflammation of some organ is not a cause, effect, or concomitant circumstance, must, we think, be at once acknowledged. Indeed, the very generality of the prevalence of inflammation, and the great variety of conditions under which it manifests itself in the economy, have led celebrated observers, as Andral, Magendie, &c., to repudiate the term as vague and inexpressive. Certain phenomena, however ill-expressed, are well-understood by this word, that are by no means represented in Andral's term *hyperæmia*, which labours under the disadvantage also of expressing a state which may exist independently of inflammation. "Hence," say Drs. Alison and Bennett, "it has been the object of British pathologists to give precision to the old term inflammation, rather than change it for another, perhaps more unsatisfactory." Investigations into the intimate structure of the various organs, and the anormal processes they are the subjects of, have been wonderfully forwarded in our times by means of the microscope, whose improved construction necessarily preceded any great advances in this direction. "It is remarkable, and at the same time conclusive of the soundness of Mr. Hunter's doctrines of inflammation," says Mr. Travers," that the additions thus made to the illustration of this great subject of his researches, though materially affecting details, so far from invalidating, tend strongly to confirm the general principles which he has laid down, to carry out the views which he entertained, and to embody those which he left in shadow."

We have to apologize to our readers for having left Mr. Travers' book so long unnoticed; the productions of his pen have been few but valuable, and anything original in this age of book-making compilation is indeed acceptable. The present work is not likely to diminish his reputation, giving, as it does, an able description of the various phenomena attendant upon inflammation, and a succinct account of the progress of the healing process observed under the microscope in many experiments. Dr. Bennett, in the pamphlet, the title of which we have also placed above, endeavours, and we think with great success, to point out in a more distinct manner than has hitherto been done, what are the essential phenomena of inflammation, and to show the connexion of these with a perversion of nutrition, explicable by the cell theory. To present a complete analysis of works themselves so condensed would not be a very profitable labour, and we shall therefore notice only in each such portions as present most novelty and interest.

*The Capillaries.*—Upon these Dr. Bennett offers some interesting observations. Blood-vessels examined by the naked eye seem to consist of but three coats; but Henle has shown that in fact they are distinctly divisible into six layers, of each of which the intimate structure differs in arrangement. These coats diminish in number as the vessels approach the periphery, but even very minute ones continue to possess three of them. At length, in the true capillary or intermediate vessel, only one exists, consisting of a firm, transparent membrane, without the smallest opening, and thickly studded with variously-shaped nuclei.

"In the numerous demonstrations I have made of these structures, I was struck with the resemblance which the capillary vessel presented to the fibres of non-voluntary muscles. I subsequently discovered that, if the mucous coat be carefully removed from a portion of intestine, (say in the rabbit,) and the remaining structure rendered transparent by acetic acid, a longitudinal and transverse coat, with longitudinal and transverse lines, formed by elongated nuclei, are apparent as in the coats of arteries. In short, the two structures in some demonstrations were so similar, that I should have had some difficulty in recognizing one from the other, were the microscopic examinations alone attended to. Repeated examination of this fact, as well as numerous observations on the organic muscular fibre, have led me to the conclusion, that no structural difference exists between what is called the muscular coat in the intestines, and that constituting the third and fourth layers in arteries, as previously described. Neither does any essential difference exist between these last and the ultimate texture of the capillaries. If this opinion be correct, then it will be natural to suppose that their function is also similar.

"The term muscular fibres, as applied to this structure, gives rise to very erroneous notions. Persons naturally conceive that some relation exists between voluntary and non-voluntary muscle, whereas, no two elements can be more distinct. The fibres seen running longitudinally and transversely, as on the intestinal coats, the fibres of the bladder, stomach, &c., have no analogy whatever with the striated fasciculi of voluntary muscle. Their mode of contraction, also, is perfectly unknown. The fact, however, viz. that the structure hitherto called non-voluntary muscle is the same in the contractile coat of the muscles and intestines, the middle coat of the arteries and in the capillaries, leaves very little doubt in my mind, that the vital contractility possessed by all these tissues is the same, and dependent upon the same causes." 26.

Of the *Functions* of the capillaries it is observed—

"The more specific office of the capillaries and intermediate vessels is evidently, 1st, so to subdivide the blood that it may reach every portion of the organism, and enable its corpuscles to perform their function: 2d, to offer a membrane by means of which exosmosis and endosmosis may be effected.

What the connection may be between the vital properties of those vessels and the exudation of blood-plasma it is difficult to determine. We may remark, however, that the delicate homogenous, structure they present, admirably fits them for acting as fine filters subjected to vital laws, retaining the solid corpuscules and granules, and allowing only the fluid portions to transude. How far the circulation is influenced by the contractility of the capillaries is still a matter of inquiry.

"From these considerations, the importance of the capillaries, as connected with nutrition, will become apparent. So long as they only permit that amount of blood-plasma to exude which is capable of supplying the quantity dissipated by waste, so long they may be considered as performing their functions in a normal manner. But when circumstances induce such a change in them that the amount of exudation is materially diminished or increased, then an *anormal* state is occasioned. If the amount be diminished, atrophy will be produced, if it be increased, that peculiar pathological change, hitherto denominated *inflammation,* is constituted."

*Direct Effects of Stimuli and of Wounds.*—Mr. Travers presents the following brief summary of appearances observed, and which were alike produced whether the experiments were performed by the application of any stimulant substance, as salt or ammonia, or the infliction of a wound.

"The first effect of a drop of stimulant fluid, or of a wound upon a transparent web (frog's foot), as seen in the field of a powerful microscope, is to arrest the circulation at the part. Around the point of absolute stagnation the column of blood oscillates, and the particles are seen to separate and congregate in small irregular masses, presenting varieties of shape, some being perfect ellipses, others spherical. The vessels are dilated, and in proportion their fulness is increased and their pink colour heightened. Still more remote from the stagnant centre, increased activity of circulation prevails. The point of stagnation—the very slow circulation in the part immediately surrounding it, the current still oscillating in parts—and beyond this the more rapid and vigorous circulation, are manifested for several days. The contrasted appearance of one portion of the web stationary, and another in brisk circulation, is striking."

In a period varying from ten to twelve days this condition of congestion of the parts disappeared, and the circulation became established over the whole web.

*Theory of Inflammation.*—Where the stasis of the circulation, however, is produced by a more considerable irritation, the distended vessels relieve themselves, either by rupture and consequent hæmorrhage, the effusion of serum, or the exudation of the *liquor sanguinis* or plastic element of the blood. Dr. Bennett attributes these early phenomena of the inflammatory state to a vital contractility and consequent relaxation of the capillaries, analogous to, if not identical with, spasm and paralysis of muscle; and to an increased attraction between the corpuscules of the blood and the surrounding parenchyma, by virtue of which these approach from their natural position in the centre to the sides of the vessels.

He also regards the *anormal exudation of the liquor sanguinis or blood-plasma as the essential phenomenon of inflammation.* That *effusion* frequently occurs independently of the inflammatory process is familiarly known; for example, in various forms of dropsy of the limbs. It may also precede or accompany the true inflammatory exudation, but is quite distinct in its nature. "The effusion of serum under compression," observes Mr. Travers, "œdema, or lesion, devoid of blood particles, as in sprain, is to be distinguished from the effusion of *liquor sanguinis* in wound and inflammation:—the former being the albumineo-aqueous and saline compound, forming the serum of the blood, the latter, the fibrinous constituent separated either from the effused *liquor sanguinis*, or within the inflamed vessels; the last alone being susceptible of permanent organization.

Dr. Bennett thus too endeavours to account for the distinction.

"Why it should happen that venous congestions are never accompanied by an exudation of blood-plasma, whilst arterial congestions are, is a point that no one yet has endeavoured to explain. To me it appears certain that all inflammatory effusions occur through the capillary or intermediate vessels, and not in such vessels as may properly be called arteries or veins. The vein is a very compound structure, and when distended to the utmost only permits the more fluid portions of the blood to pass through. The capillary vessel, on the other hand, is a most simple structure and exceedingly delicate, so that when distended we may readily understand that it admits not only the serum but the more inspissated *liquor sanguinis* to pass through also. But it is scarcely possible to suppose that the mechanical difference in the tenuity of the filtrating membrane should constitute the only distinction. It is impossible to reconcile the phenomena without having recourse to some active vital power of attraction between the blood and parenchyma, as formerly explained; a power which, operating in the one case and not in the other, causes different constituents of the blood to become exuded. We are compelled, in all our considerations of the subject, to go back to this explanation, as to an ultimate fact." 39.

The exudation of *blood-plasma* is then the only positive proof we have of the existence of inflammation; and the want of a recognition of it as the essential phenomenon has surrounded the descriptions of the disease with vagueness and inconsistencies. Thus, even in Mr. Travers' present work, he enumerates heat, redness, pain, and swelling as the local symptoms of inflammation, any one or more of which may yet be absent; and when present serve in part rather to characterise the preceding congestion than the inflammation itself; while he treats of the exudation merely as one of the effects or results of inflammatory action. A confusion of ideas upon the subject has led also Dr. Macartney to state that the presence of inflammation is opposed to the healing of wounds, while Sir A. Cooper maintains that this is impossible in its absence.

*Constitutional Symptoms or Effects of Inflammation on the System.*

We transcribe some of Mr. Travers' excellent observations upon this subject.

"When the local inflammation is in such organs or of such amount as to rouse the system, this partakes (?) of the inflammation. The blood undergoes changes in passing through inflamed vessels which affect its natural properties. The brain and nerves become preternaturally excited by it, the functions of the entire digestive apparatus and of respiration are hurried and imperfectly performed; consequently the excretions are altered in quantity and in quality, and the vitiation of the blood increases from the suspension of the relief derived from its habitual and required issues; for what the blood in its circulation parts with, is as essential to its health as what it gains. Meantime the inflamed part undergoes changes very typical of those which the system indicates. No approach towards healing is ever seen in such a state of the system. The degree of fever, the seat and the symptoms of the inflammation must be taken as the index and measure of the changes which are wrought in the part. If the adhesive or granulating process has not commenced it is prevented; and if it be in progress it is destroyed. The ulcerative process becomes utterly indolent and ill-conditioned, or it passes into gangrene. The changes, more or less, are all on the losing side. The whole system is inflamed (?), and the local malady and its cure are merged in the general malady and its treatment. It is almost ludicrous to observe the nice distinctions and refinements of the ancient, and even of some modern surgeons, in the selection of local applications, when the parts take so subordinate a share of the disease, or are so far beyond reach of treatment by local remedies, as to be scarcely susceptible of good or harm. It is like galvanising the muscles to restore life to the exanimate system.

"In inflammations which are set up by grave injuries, the system, if not at once disabled or in some way disordered along with the parts, is frequently predisposed to an earlier sympathy, induced by the shock which it has at the same time sustained. This sympathy constituting 'irritation,' is often of a more dangerous description than inflammatory fever, as I have elsewhere fully explained.*

"On the other hand, many inflammations having a very slight beginning, as regards their exciting cause, seem to owe their unmanageableness, and rapid transition from bad to worse, to a previously diseased or worn-out habit of body, and the typhoid diathesis prevailing in such circumstances. Some indeed originate from this cause independent altogether of local injury—such cases are always of serious aspect. But in favourable circumstances, of which youth and temperance are the foremost, the sympathetic inflammatory fever gets up gradually even after severe injuries, and evinces no more than what must be regarded as the unavoidable, nay the gratifying proof of universal and salutary consent between the whole of its parts; and the career of inflammation towards the accomplishment of the purposes to which it is instinctive is but little retarded." 56.

Among the remarks upon *Blood-letting*, we select the following:—

"The choice of measures, *i. e.* local or general blood-letting, is determined, partly by the relation of the parts affected to the centre of the circulation, and in part by the more or less urgent necessity that exists for disembarrassing the general functions, and arresting destructive inflammation. In visceral inflammation venesection is indicated and warranted to the utmost extent that the powers of life will bear; for here the mass of blood is so altered and spoiled for its proper and healthy purposes by the direct implication of the blood-making and blood-preparing organs in the disease, that relieving the system of its presence to the extent that can be borne, is the main resource we possess for its preservation. As we would remove a poison, a *materies morbi*, in such cases we take away blood. Its altered condition is palpable when eliminated from the body; it undergoes a peculiar separation of its parts, and presents other appearances not manifested by healthy blood. The difference of the blood within the vessels from that of health, is not less, if we could fully appreciate it. If inflamed blood be transferred into the vessels of a healthy animal of the same species, as his own is withdrawn, instead of supporting, it rapidly destroys life. A freer circulation through the smaller vessels, and those of the excretory glands especially, ensues

---

* Inquiry concerning Constitutional Inflammation, 1827.

almost immediately upon a full blood-letting: the sense of overwhelming oppression is relieved, and the inflammation, if not abridged by its effects, is disposed to a kindlier termination.

"There are two false doctrines concerning blood-letting for inflammation, which cannot be too strongly condemned: the first, anticipatory blood-letting, by which I mean, the large and repeated detraction of blood before inflammation, being considered inevitable, has actually manifested itself—on the hypothesis of starving the action, and thus rendering it tractable—which is a direct attack on the vitality, and fatally prevents the action, if it do not destroy the resisting powers of the system. The second, continuing the employment of the lancet so long as the last-drawn blood exhibits the signs of inflammation, which if drained to the last drop it would do; or, in other words, not reflecting that there is a line beyond which the practice becomes destructive instead of remedial; and that there are many inflammations which do not admit of arrest by depletion, and upon which other modes of treatment are efficient to this end, even though not an ounce of blood be drawn. Many lives have been sacrificed to the prevalence of these irrational and absurd notions, and many preserved in their extremity by being fortunately placed beyond the reach of the surgeon; especially, I am induced to believe, in military practice." 59.

*Terminations or Results of Inflammation.*—Dr. Bennett having shewn that inflammation consists essentially in an exudation of the *liquor sanguinis* or blood-plasma (*i. e.* the fluid portion of the blood composed of fibrin dissolved in serum), next adverts to the changes this exudation may be subjected to: *i. e.* the *terminations* of inflammation.

"What have hitherto been considered as the terminations of inflammation, viz. adhesion, softening, induration, suppuration, granulation, ulceration, gangrene, &c. modern pathology has shown to be in themselves highly complicated processes. John Hunter was of opinion that these various results were explained by supposing the inflammation to undergo certain modifications, or that it terminated differently according to certain tendencies which it possessed; hence the terms adhesive, suppurative, ulcerative, and gangrenous inflammation. According to the view, however, which we have endeavoured to establish, viz. that inflammation essentially consists in an increased exudation of blood-plasma, it follows that this process in every tissue and under all circumstances is the same. The exudation having once taken place, any further changes we may observe in it will depend upon the amount or rapidity with which it is poured out, the tissue in which it occurs, its chemical and vital proportions, and the accidental circumstances which modify or destroy growth both in the vegetable and animal world. Whenever the exudation of blood-plasma poured out coagulates, it can only be removed from or assimilated to the economy by one of two results, viz. by death, or by passing into organization. When it does not coagulate, which is an occurrence of great rarity, it may again pass into the vessels by endosmosis, and in this way the parts return to a normal state. Resolution, however, as at present understood, is a very compound process. It is used to express the disappearance of the inflammation without its producing any external lesion. In this manner it may follow hæmorrhage, softening, or suppuration, and great difficulties impede our attempts to ascertain with exactitude how, under these circumstances, the exuded and organized blood-plasma is absorbed. For this reason, therefore, we shall discuss this portion of our subject in the following order—1, the Termination of Inflammation in Death of the Part. 2. In Organization. 3. In Resolution." 44.

We regret that our space will not allow us to follow the author into the details of this subject: we may, however, present a brief sketch of the conclusions he arrives at, for the purpose of exhibiting his application of the doctrine of Cytogenesis to the explication of the various phenomena resulting from inflammation. The nature of the cellular theory of Nutrition, which has accomplished so much in simplifying and generalizing our knowledge of the intimate structure of animated beings, has already been explained in this Journal.* As the solid, coloured, corpuscules of the blood are subservient to the maintenance of the animal heat of the system, so is the fluid portion, the *liquor sanguinis,* the essential element of nutrition, the *plasma* destined to supply the material of every variety of structure. In this, "*nucleated cells* are formed, which are again ultimately developed into the different textures, or made subservient to the function of secretion." When the exudation of this blood-plasma takes place in a defective degree, we have *atrophy* induced; but when, on the contrary, from the distended and attenuated state of the capillaries, it exudes in excessive quantity, *inflammation* is said to be present: and the nature of the termination of the inflammation depends upon the changes which are wrought in this excessive exudation. Why these should differ in different cases is not always explicable, any more than why, from the same plasma in its normal condition, so great varieties of ultimate structure are evolved. The termination of inflammation in the *death of the part,* is termed *mortification* or moist gangrene when acute, and *ulceration* when chronic. Of the former Dr. Bennett observes:—

"Occasionally a very large amount of blood-plasma is thrown out, constituting a violent inflammation: a greater or less number of capillaries are also ruptured and blood-corpuscules are more or less mixed up with the *liquor sanguinis* exuded. The exudation thus formed compresses the part so as to obstruct the blood-vessels, and prevent the continuance of any circulation in it. Under these circumstances, instead of forming a blastema for the production of new organisms, it undergoes chemical changes which induce in it decomposition, and the part is said to be mortified."

Mortification arises from various other circumstances besides inflammation, as the effect of different chemical or mechanical injuries in directly destroying the tissues, or the leading vessels supplying the part, the obstruction of the arteries in the aged, &c. Examples of mortification from inflammation are seen after burns, exposure to frost, some forms of erysipelas, &c.—the excessive amount of exudation from all these causes obstructing the circulation.

In *ulceration* there is less exudation thrown out than in gangrene, which neither undergoes decomposition or organization, but acting as a foreign body upon the circulation of the surrounding parts, thus causes their death. Examined by the microscope it is found to contain numerous minute granules, mingled with some imperfectly-formed cells. It, and the structures compressed, eventually become broken-down into a semi-fluid mass, which obtains exit at the surface.

*Termination of Inflammation in Organization.*—Organization consists in

* See Med.-Chir. Review, Vol. 40, p. 1, Vol. 41, p. 124.

the formation and development of nucleated cells, and, in the great majority of cases, the blood-plasma exuded in inflammation forms a *blastema* or germen for their production. Under different circumstances, however, various degrees of such development occurs; and the author enumerates four varieties of modes in which organization may be effected, in each of which the microscopic characters of the cellular resulting production differ. 1. From the exudation may be formed certain *plastic* corpuscules and primitive filaments constituting *lymph,* as seen on the surface of the serous membranes. 2. It may be transformed into what the author terms *exudation granules, masses and cells,* forming the inflammatory *softening* of parenchymatous organs. 3. It may be transformed into corpuscules (pus) constituting *suppuration.* Purulent matter does not therefore, as supposed by Hunter and his followers, result from an altered action of the vessels, secreting it from the blood: but is a fluid having its own distinct nucleated corpuscules floating in the *liquor puris,* and results from changes which have been operated in the exuded plasma.

"Before quitting this subject it is necessary to observe that the three varieties of corpuscules we have described frequently undergo important modifications. What has been said refers to what may be called the type of such formations. In individuals who labour under particular cachexies, as scrofula, syphilis, scurvy, &c. the corpuscules vary more or less in their characteristic properties. We believe, however, that the separation of the three kinds of corpuscules, which distinguish the three great products of inflammation, will facilitate the future study of the differences which these structures occasionally present. This the more so, as, according to our observations, it is rare, that the additions of re-agents will not enable us to refer them to one or the other of these divisions."

4. *The exudation may be transformed into permanent tissues.*—The re-generation of lost parts is accomplished or some substance is produced in lieu of them. Complicated tissues are seldom regenerated, as the skin, lungs, brain, &c. but simple tissues are, as the filamentous, fibrous, and even the osseous. Of all reproductions the fibrous is most frequent. This new production of tissues from the plasma is accomplished by the formation of nucleated cells, exactly as is observed in the organization of the various tissues of the embryo. As an example of what occurs in other parts, *mutatis mutandis,* we may select the description of the processes by which wounds are united.

"After a time, which varies according as the blood more or less abounds in fibrin, the cut surface is glazed, as it is called, that is, the exudation has coagulated on the surface, and is transformed into plastic lymph. If now the parts are accurately brought together, little more exudation takes place. Cells are formed, which rapidly pass into a fibrous formation, and healing, or *union by the 1st intention* is the result. When, however, the lips of the wound remain apart, or there has been loss of substance, the exudation is more copious externally than internally. The portion which is infiltrated into the tissue surrounding the sore, constituting the inflamed, red, and indurated margin, is transformed into exudation-cells, which, on breaking up, are absorbed. Some of these occasionally find their way to the surface, and become mixed with pus-corpuscules,—a circumstance which probably, with some, has supported the supposition that these structures are different stages of one growth. On the other hand, the exudation which is poured out externally on the surface of the sore is very abundant, and is transformed partly into the pus cells, and partly into primary cells, which are, by the process of development, converted into fibres, and ultimately constitute the cicatrix. The portions of exudation which are undergoing this process are called *granulations*. As these become more numerous, the amount of pus diminishes, and a greater tendency is manifested in the exudation to pass into permanent tissue. At length pus ceases to be produced,—the whole exudation passes into fibres, a new surface is formed,—the which, contracting, after a time, constitutes a cicatrix." 62.

Why from the same plasma exuded into the inflamed tissues these particular transformations are produced is not easy to explain ; but there are certain circumstances which exert considerable influence in determining the particular kind of change, and of these Dr. Bennett mentions three. 1. The nature of the surrounding elementary structure. Thus, exudation in the neighbourhood of cellular tissue is developed into cellular tissue, and that near bone into bone; while every tissue is liable to hypertrophy, during chronic exudation. 2. The degree of vital power of the organism exerts much influence. Hence the imperfect organization of the exudation when the vital powers are low, or the constitution of the blood is changed. 3. Great influence is also exerted by the rapidity with which the exudation has taken place. If the inflammation has been acute, and the exudation large, one of the three forms of corpuscules, the exudation, plastic, or pus, is readily produced ; but if it be chronic, and the exudation comparatively sparing, the higher degree of development of the cells into fibrous or other tissues, or the hypertrophy of various organs, results.

*The Termination of Inflammation by Resolution.*—Resolution or absorption of the exudation may follow any of its transformations, with the exception of that which has converted it into permanent tissue. "The early phenomena first disappear ; the capillaries recover their contractility : the attraction between the blood and the parenchyma ceases: and the blood within the vessels begins to oscillate, and at length flows in a continuous stream." Occasionally, but rarely, the plasma remains uncoagulated for some time after its exudation ; and then, at the restoration of the natural condition of the vessels, may re-enter them by endosmosis unchanged. Once coagulated, it cannot be absorbed, unless again rendered fluid. The nucleated cells are broken up, and the transformed exudation reduced to a soft, pultaceous, diffluent substance.

"The disintegration of pus-corpuscules previous to absorption is evidently favoured by the pressure which the abscess receives from the contraction of the filamentous and elastic tissues that form its walls. This is shewn by the increased hardness which always accompanies the disappearance of suppuration by resolution, and the good effects which result from direct pressure employed by the surgeon to discuss these swellings. In Berlin the most successful treatment for bubo consists in simply placing a stone of one or two pounds' weight over the tumor in the groin, as the individual is laying on the back. It is probable, also, by increasing the contraction of the integuments, as well as by removing fluid from the neighbourhood of the part, that irritants, blisters, and cauteries, are beneficial in the resolution of abscesses."

The molecular fibrine which re-enters the circulation after the breaking-up of the exudation is for the most part eliminated by the kidneys.

H 2

Schönlein, Zimmerman, and other German physicians, have especially called attention to the turbid and highly coagulable condition of this fluid during the resolution of inflammatory diseases.*

Mr. Travers takes a very different view to Dr. Bennett of the various results or processes of inflammation; and describes these under the separate heads of adhesive, suppurative, ulcerative, and gangrenous observations. As illustrative of the nature of the healing process he gives a complete description of Dr. Todd's dry preparations, now in the Museum of the College of Surgeons: but, as these, owing to the absence of any accompanying memoranda, left the subject very incomplete, he was induced, with the aid of Mr. Quekett, to repeat the various experiments upon the web of the foot of the frog, allowing the animals to live, and witnessing, and recording from day to day, the various stages of the healing process. These records will prove very valuable aids to future observers, as their accuracy can be relied upon. We can only allude to the general summary of results, advising all those who feel interested in the subject to consult the detail of the various phenomena.

The first result of local irritation is *stasis* or arrest of circulation, proportionate in extent and duration to the amount of injury done. The circulation is oscillatory at the verge of the stasis, beyond this preternaturally slow, and yet farther off rather quickened. 2. Effusion of serum or of *liquor sanguinis*, the latter denoting the severer degree of irritation. 3. Fibrine of the liquor sanguinis is only capable of organization when separated from other constituents of the blood, as the coloured globules, &c. 4. In the act of coagulating upon the face and sides of the wound, the fibrine separates from the serous portion of the liquor sanguinis, and becomes a membranous crust, covering in the wound at all points, the sections of vessels, &c. forming the intermedium for vascular anastomosis in union by adhesion. 5. The separation of the lymph particle from the blood within the vessel at the margin for supplying the permanent plasma. "The first deposit effused with the liquor sanguinis is an amorphous exudation, and presents no such regular figure and arrangement as the lymph particle which has been separated within the vessel before deposit." The first serves as a mere temporary bond of union, and nidus for vessels, and is then absorbed, the second is a substantial addition of structure. 6. The oscillation attending the recovery of the circulation is the first movement towards the formation of a new circulation. 7. A blood-corpuscule escapes at several points of the margin of junction between the original circulation and the new plasma. Its track is rapidly followed by a file of single corpuscules. After a while, motion, at first oscillatory, and afterwards progressive, is imparted; a distinct appearance of vessels is conferred, and numerous anastomoses occur, until the free margin of the lymph more and more encroached upon is at last crossed and covered. Isolated or independent centres of vascularization, unconnected with the original capillaries, do not exist, although appear-

* See Med.-Chir. Rev. Vol. 41, p. 150, for an account of Zimmerman's researches upon this subject.

ances sometimes resemble these: the fabricator of the new vessel is the blood-corpuscule derived from the adjacent capillary.

"The temporary stasis seems to be necessary to the exudation of the liquor sanguinis, its continuance to that of the separated lymph particle; and not less the graduated impulse of the returning circulation to the elimination of the blood-corpuscule in single globules for the fabrication of new vessels: for if it were in mass (hæmorrhage) it would destroy instead of promoting organization.

"All capillaries, then, may convey either pure serum, or the liquor sanguinis, or the blood-corpuscules, severally and distinctly; secretion or separation being, like all the processes of inflammation, a positive action subject to vital laws, not an accidental outpouring, like that from a ruptured vessel.

"I greatly doubt if the separation of the lymph particle within the vessel is ever a normal state, *i. e.* unpreceded by stasis, although breach of texture is not necessary to its appearance, an exudation of organizable lymph continually happens without primary breach. The intrusion of the blood-corpuscule into the serous capillary is only under the nisus which leads to congestion, or that of inflammatory action. When the last is established, colourless vessels come into view, becoming at first tinted, and then deeply coloured. The lymph is laid down as the arterial capillary circulation resumes, and always of necessity precedes the generation of new vessels; which process does not commence until after the original circulation, to the extent that is uninjured, is fully restored, as an energy beyond that which exists in the normal state is required." 170.

To the question whether *effused blood is instrumental in effecting adhesion,* Mr. Travers thus replies.

"Is the blood, when effused from wounded surfaces, a medium of organized adhesion? or capable of becoming so? I answer in the negative; the question turns upon a delusion. If the wound be so small as that the effusion of blood is restrained by the adaptation of its sides, whether naturally falling together, or artificially compressed, the separation of its colouring matter is shewn by a plentiful oozing of sanies at its mouth, and the formation of a crust. If, on the other hand, the wound be of such form or size as to prevent coaptation, or be attended with loss of substance, the coagulum of coloured blood being in proportion, acts as a foreign body, and must be dislodged prior to healing. Hence the difference in the time, and often in the mode, of healing of a small gaping wound left to itself, or a wound with loss of substance, and that of a larger wound, whose sides are immediately brought and maintained in contact. Thus the agglutination of the lips of a small wound by a thin layer of blood, a merely temporary expedient, is no bar to the union, but the contrary, both in respect of hæmorrhage and union, though never forming the permanent bond. In truth, no wound of any dimensions, however favourably situated for the adhesive process and rapidly united, has not, when fresh, a layer or pellicle of blood coating its surface; not admitting of removal by abstersion, but insusceptible also of healthy organization. The separation and deposit of fibrin takes place distinctly and after an interval. This is marked even in cases of simple division of the solid, but in loss of substance occupies many days: being step by step, and only just a-head or in advance of vascularization. No preparation so exactly illustrates the temporary use, and the permanent non-effectiveness, of the clot of blood; and, on the other hand, the distinctness of organizable fibrine destitute of colour and available for union, as the wound of an artery by a round ligature. The ultimate complete absorption of the clot is equally well exemplified." 82.

*Of Suppurative Inflammation Granulations, and Pus.*—Mr. Travers regards pus as a secretion, the lymph being organized into granulations for its production, where loss of substance has to be provided for.

"This consists in the formation of granulations or small eminences, into which the lymph pellicle, common to all wounds, is elevated, so organized as to constitute a temporary or occasional apparatus for the secretion of pus. The granulation is a mesh of the terminal loops of capillary pencils, formed, as has been described, under the adhesive inflammation, in the newly-deposited fibrinous membrane coating the surface of the breach or cavity in the original solid.

"The aspect of the suppurating membrane also varies to such extent as scarcely to exhibit, in some circumstances and situations, the granular form; *e. g.* upon the walls of abscesses, and upon the free surfaces of mucous and serous membranes: but the fibrinous bed and the capillary loop of new formation, and a corresponding alteration of the pus-secreting surface from its normal state, will always be detected upon careful examination, being essential elements of the suppurative process. The product of granulations is a yellow, unctuous, inodorous fluid of a cream consistence, which is necessary to their function, and not less to their existence, until the purpose of their formation, viz. their coalition and levelling, is accomplished by the free anastomosis of their proper vessels; and the new substance, be it skin, muscle, membrane, or bone, is perfected. The granulation is hollow or cellular when first produced, and the secretion of pus within it is progressively substituted by the secretion of the material required, which gives it the permanent figure of the new solid; the pus always diminishing in proportion as organization proceeds, until the secretion of pus ceases altogether, and the granulation properly so-called disappears." 111.

In another part of his work the author thus states his theory of the formation of pus.

"Pus, I believe, to obtain its characters of consistency, opacity, and colour after exudation, and to consist of the superfluous or waste lymph which has been separated during the adhesive stage from the mass of the blood, held in solution by the serum, being thus a chemical modification of the constituents of the liquor sanguinis: in short, the latter fluid deprived of its original characters and property of spontaneous coagulation. Pus particles resemble those of lymph seen in the vessels under inflammation, except that they appear broken-down and partly dissolved in their texture instead of compact, and of less regular figure; and if, when suspended in a drop of fluid, compared with the elastic blood-corpuscule, to which they bear no analogy whatever, utterly inert and devitalized. We never see pus in the blood-vessels but in fatal phlebitis, and if introduced into the circulation by injection, it is destructive to life. Although, therefore, a clean-wiped granulating surface soon presents a covering of pus, it is exuded as a colourless fluid, of a more dense and unctuous consistence than serum. Its appearance is simultaneous with the disappearance of the lymph particle from the veins. The suppurative action being determined, or in other words, the separation of the proper lymph particle put an end to by its sufficient deposit in granulation, and the inflammatory nisus still prevailing from the continuance of the irritation—for no imperfect state can be perpetuated—the superabundant lymph particle, at no time coloured, along with the permanent fluid or serum of the blood, is strained off through the pencils forming the terminal loops of the granulation. Thus is obtained the two-fold purpose of relief to the loaded capillary circulation, and a bland and homogeneous protecting fluid for the granulation, during the period of its growth up to that of final organization. When the rudimental fibrine is no longer needed for the new structure, it is used, as in nature all remnants are, for a new but not less important purpose, the preservation of that structure. Pus is as necessary to the maintenance of granulation as lymph was to its formation. But a change is necessary to fit it for its new function, and this is provided for by a new arrangement or a new action of the secreting capillaries, and a chemical change, which destroys its vital property and amalgamates the separated lymph-globules with the serum of the blood. The precedence of adhesive to suppurative action is sufficient to render presumable a necessary connexion between the lymph separated during the first process, and afterwards disappearing, and to explain the invariableness of this relation in the order of their appearance. There is no analogy between the effusions of serum or of liquor sanguinis incidental to primary wound or injury of any kind, and pus, yet the ingredients of the two latter are the same: it is by the combinations of a vital chemistry that their appearance and sensible properties differ, and this we are incapable of imitating.

"If this theory be admitted, it will explain the appearance of pus in the absence of the especial granular structure or distinct pyogenic membrane, as seen upon mucous, serous, and synovial surfaces and canals; and even in the absence of fibrinous exudations, as in certain modes of inflammation, where the habits of the parts, or the character of the inflammation render them incapable of carrying on the adhesive action, or that action is by violence interrupted." 174.

The several stages of the action of a blister, according to the existence of a slight or large amount of irritation, present an illustration of the different inflammatory processes. Thus, there may be the mere aqueous effusion under the epidermis; the jelly blister abounding in albumen and fibrine and slow of healing; the production of pus; ulceration; and even gangrene.

Pus then is *always an inflammatory product;* and where collections are found unaccompanied by marks of present inflammation, there are still always sufficient vestiges, in altered structure, to demonstrate that it has existed, and that in the *adhesive* form.

"The suppuration in diffused cellular inflammation, in cold abscesses after typhus, and other cases of constitutional exhaustion, as especially erysipelas and animal poisons, are the cases which appear to authorize the belief that pus may be formed independent of adhesive inflammation. But it is my belief that, in these cases, the effused fibrine dies before its organization is accomplished, as granulations perish in certain ulcers. The vitality of the blood is so low as to render it unfit in quality for organization, or the circulation is so feeble as to be incapable of organizing the deposit; and thus the adhesive process is either imperfectly performed, or, is destroyed by the counteraction of ulceration, or by gangrene. The case is equivalent to that of ulcer in which granulation is wanting and cannot be produced, or if produced, maintained, owing to the preponderance of the absorbent over the depositing action, or the failure of all action. And in confirmation of this opinion are the sweeping extent and devastation of these abscesses, the bad quality and early decomposition of the pus they secrete, the frequent escape and mixture with it of blood and of gas, and the enormous sloughs of cellular membrane and fibrinous deposits by which they are accompanied. It is therefore no pathological exception, unless indeed an exception establishing the rule, since the same order of actions is preserved, but the constructive are cut short or overpowered by the destructive, from the diseased state of the blood and of the general habit. * * * * * * * The phenomenon of late years brought under the notice of pathologists, of what are called *secondary deposits,* and of which I have seen many examples, require only to be referred to here. So far as the question, whether inflammation be indispensable to the production of pus, bears upon such cases I consider them to form no exception to the universality of this law, and consequently repudiate the accounts of insulated deposits, unless in cases of admixture of pus with deposits of fibrine and coagula of blood, as in inflammation of veins, which I have seen both in the human subject and in animals, the subjects of experiment." 117-119.

Depositions of lymph in various organs, when occurring in small quantities, may be easily mistaken for pus. "The theory of the consentaneous or consecutive occurrence of these deposits of lymph and abscesses, in parts remote from the seat of inflammation, is a distinct question, and to be so treated."

*Ulcerative Inflammation.*—In this chapter Mr. Travers replies to the various objections which have been offered to Hunter's theory of the agency of the absorbents in ulceration. He justly observes that, although no direct proof of such action were adduced, as applied to the products of inflammation, yet the agency of the absorbents being admitted as regards the removal of the *debris* of sound structure, the *onus probandi* of their not being so employed in broken structure rests with the objectors. Among the objections offered, the author notices the following.

1. *Ulcers spread most rapidly during inflammation, when absorption is diminished.*—Here the different stages of inflammation are confounded. In the early stage, when inflammatory fever is present, absorption is diminished; but it is augmented in the latter, when hectic is present. Ulceration cannot exist unpreceded by inflammation, and, when we wish to augment absorption, we first augment the activity of the capillaries by local irritation, or excite irritative fever by the administration of mercury.

2. *The tissues best supplied with absorbents do not ulcerate so readily as others inferiorly supplied. Thus bone, which is quickly absorbed before an aneurism, does not ulcerate so readily as cartilage which is very slowly absorbed.*—Here interstitial and ulcerative absorption have been confounded; the former being quite independent of inflammation, and many textures are prone to both forms, the mode, and degree of irritation determining which shall prevail. Skin and mucous membrane of all textures, best supplied with absorbents, are also those most prone to ulceration.

3. *The state of congestion favourable to ulceration is adverse to absorption.*—It is so to healthy absorption, but, as it is a strong predisposer to inflammatory action, its establishment is consequently always looked upon as a probable fore-runner of ulcerative absorption.

4. *Granulations ulcerate more rapidly than cicatrices, though they cannot be better supplied with absorbents.*—The granulation is in an unfinished stage, the cicatrix is a complete structure. The superior absorbing powers of the granulations may be judged of by the application of any minutely divided substance.

5. *A part must become fluid before it can be taken up, and why should it not then pass off with the discharge?* In open ulcers much must pass off in this way: but, what becomes of the discharge in extensive caries of the vertebræ, or where large collections of matter are disposed of? "I have seen cases in which pints of matter have been taken up from psoas abscess, and the parietes recover without breach."

"The rapid and extensive occasional action of the absorbents in cases of inflammation without breach, as the removal of the serum in local dropsies, the lymph in iritis, &c. is as incontestable a phenomenon as their continual action to regulate the adjustment of solid and fluid by removing useless and decayed parts in the healthy economy; and when inflammation is attended with breach, constituting ulcerative inflammation, they are the sole organs of the removal of parts, and no animal structure is exempt from their operation. They do not take the initiative in inflammation, but act subserviently to prior changes. Ulceration, when it occurs, is consecutive to adhesion and suppuration, in almost all cases; and although suppuration may now and then pass without ulceration, in the same manner as adhesion prevents suppuration, yet the frequent case of ulcerative inflammation succeeding to abscess, and the very rare existence of ulceration without pus, constitute the ulcerative, third in order, of the processes of inflammation." 187.

The following are valuable practical observations:—

"Although 'ulcer' is a comprehensive name for sores formed by the ulcerative inflammation, in many states and phases of their existence such sores exhibit no ulcerative action, as when that mode of inflammation is arrested. The interstitial absorption is the process by which what is called a healthy (healing) ulcer is dressed, levelled, and prepared for skinning, and it is only when this natural, not morbid, action has superseded the ulcerative, that the healing process is fully established. No sore can heal while ulcerative absorption (the inflammatory process) continues either upon the edge or the centre. This mode of inflammation is so directly opposed to the adhesive, and so adverse to healing, that being arrested, the hindrance to healing is removed. To put an end to it, therefore, is the surgical indication. This is done by constitutional and local treatment jointly; in some cases the one, and in some the other plan avails most.

"To know the character of ulcers and treat them accordingly, is a subject of the first importance to the practical surgeon, and well worthy of his science, though often not so regarded. They admit of a general classification, but present many varieties and anomalies. The great distinction is the presence or absence of the ulcerative inflammation, *i. e.* the morbid condition and action of the absorbents, by which the ulcer was formed. An inflamed is an ulcerative ulcer, so is an irritable, and more or less so is also an indolent or callous ulcer: in the gangrenous or sloughing ulcer, ulcerative inflammation is, as in gangrene, a necessary remedial agent for the purpose of separating the dead from the living parts, thus clearing the surface for sound granulations, and the edge for skinning. It is not necessary that ulceration should have existed prior to granulation, although granulation must succeed to ulceration: an ulcer has no other means of repair. The adhesive, then, is the curative termination of ulcerative inflammation; and, if for any reason the part is incapable of this action, disorganization and death is the consequence.

"It is, as we have shown, by ulcerative following suppurative inflammation, that matter and foreign bodies are brought to the surface and extricated, and that preternatural openings of communication, fistulæ, and sinuses are formed. But happily the adhesive action has gone before in the majority of such cases, and consequently, both external and internal openings and passages are defended by it, and the ulcerative process guided and circumscribed. Thus abscesses point, and effusions of the secreted fluids are prevented, and preternatural communications of the viscera with the surface are walled off and shut out from the system. If sudden injury or a cachectic state of the body anticipate this adhesion, and the ulceration is undefended, the worst consequences ensue." 198.

*Cicatrization.*—This is the finishing stage of the adhesive process, and the completion of the process of granulation. It is rapid in proportion as the diameter of the ulcer is small, the surface level, and the secretion of pus diminished. A reason of the difficulty with which a sinus heals is the abundant secretion of pus from its surface. Others are its limited contact with the skin, and the non-development of granulations, which appear, however, as soon as the sinus is laid open. When an ulcer has formed upon an old cicatrix, its healing is usually tedious, owing to the defective quantity of surrounding free cellular membrane for the formation of granulation, and to the imperfect condition of the circulation in secondary formations.

"It has been a question whether skin is formed from the margin of the vessels of the skin, or upon the surface, by the vessels of the granulation. This I have no difficulty in answering. Certainly by the surrounding skin; for, first, we see it gradually advancing from the margin, and equally reducing the diameter of the sore from day to day; secondly, we never see the skinning process without the described preparation of the border, and the process is quicker or slower in proportion to the distance of the skin from the centre; thirdly, the insular patches frequently observed upon the bed of an ulcer, especially unhealthy ones, may always be traced to a portion of undestroyed skin, thus confirming the fact. Lastly, the cicatrization beneath the surface, as of abscesses and fistulæ, is not, as before observed, by the formation of skin, or of proper granulation, but by an adhesive process, which tacks the skin to the subjacent cellular texture condensed by inflammation, drawing it inwards into folds and puckers; in short, a process similar to that which we see in the obliteration of cysts by pressure, and in the formation of solid tumors, to which the skin becomes adherent during their growth. It is very common to see these folds and indents of skin over scirrhous and other tumors, having so precisely the appearance of cicatrices after an abscess or an operation, that we are erroneously led to believe one or the other must have taken place.

"The extraordinary rapidity with which an ulcer sometimes forms skin, compared with its usual rate, conveys, with other circumstances, the not incorrect impression of the vessels of the surface indirectly aiding its formation. This is resulting from the contraction of the vessels of the granulations, in their union and levelling, giving a glazed appearance to their surface as the secretion ceases, and the (quasi) pellicle begins to form. But the act of cicatrization consists not in any fresh deposit: it is simply the last stage or completion of vascularization which renders the transparent lymph surface nebulous or opaque, and this always commences from the margin whence the vessels are derived, and is progressive from the circumference to the centre. The actual form of membrane is never accomplished, such as could be separated by fair dissection at any stage, from a cicatrix: it is a permanently opake, unsecreting surface, a condensation of the new lymph with the cellular texture beneath or surrounding it, serving the negative purpose of a semi-organized covering, viz. protection to the part. Like all other new structures, it is a copy, and differs, as all copies do, whether of nature or art, from the original." 207.

Our space will allow of our making but one or two extracts from the excellent chapter upon *Gangrenous Inflammation*. Mr. Travers, after describing the ordinary forms of acute and chronic or dry gangrene, proceeds thus:

"Gangrenous inflammation may be primary, *i. e.* no other mode of inflammation going before it. The circulation of the part and neighbourhood becomes stagnant, and the vessels, both arteries and veins, are choked with broken and semi-solid coagula. Is it due to the vehement intensity of the local inflammation and consequent exhaustion of the nervous life of the part? or to a constitutional condition, which renders the solid and fluid elements incapable of entertaining the functions of the part, on which depend its temperature and principle of resistance to external agents, in other words, its life? In all cases not produced by external injury, and in many that are so produced, I believe the latter is the true explanation. * * * * * *

*     *     Gangrenous inflammation, in the larger number of cases, is secondary to intense cutaneous and cellular inflammation, and so common is this association, that the term 'gangrenous erysipelas,' and carbuncular abscess are in practical use to denote these combinations, and the distinction of gangrenous from adhesive and suppurative inflammation. It is an acute inflammation directly disorganizing, because the resisting power of the parts affected by it is inadequate to sustain the circulation: this becomes stagnant and imperfectly coagulated in the larger vessels, for the blood loses the coagulating principle as it dies, while a non-vital transudation of their fluid contents loads the surrounding cells with sanies, with which pus is intermixed, where the suppurative process has preceded the gangrenous; and the whole texture perishes and putrefies. That it is a substantive mode of inflammation is shown by its occasionally idiopathic existence, as well as its distinctly marked primary characters; by its supervention in decayed or diseased habits on very slight forms of injury; and especially by its tendency to spread along the neighbouring sound parts from the extremity towards the centre." 216.

The constitutional nature of the affection is thus stated—

"Gangrenous inflammation, therefore, is to be regarded as essentially of constitutional origin in all circumstances: its local termination is the same disorganization as follows from the work of destroying agents: in this extreme result only do the inflammation of gangrene, and the state of gangrene touch and analogize. When we speak of an inflammation passing into gangrene, we convey that the system is the medium through which such a change is expected and effected, and that thereby the whole character of the action is changed. For the process of sloughing (separation or disjunctive ulceration) to the extent necessary to clear a wound of spoiled textures, is a local process indispensable, and which excites no reasonable alarm, if the surrounding inflammation is of the adhesive kind, and the ulceration confined to the adhesive line, accompanied by a free suppuration. These are indications of tranquility of system and consequent power of the parts, whether the destruction is to the extent of a caustic issue, or involves the hand or foot. But if the spot of acute gangrene, idiopathic or from injury, be surrounded by the gangrenous inflammation, which spreads rapidly, and kills as it spreads, without provision for its arrest or its separation, we turn with well-founded alarm to the system of which it is a part, taking no comfort from the small extent or perfect reparableness, in other circumstances, of the mischief hitherto inflicted. We feel that so indicated, the failure of constitutional power is the fearful odds against which we have to contend, and this is corroborated by inquiry into the constitutional symptoms, and the pre-existing state of the patient's health and habits.

"To conclude, gangrenous inflammation, always originating in weakness, whether violence be combined with it or not, is never a process conservative of structure; and in this regard stands contrasted to all other inflammatory processes." 226.

We have perused both these books with much satisfaction and profit. The authors regard the same phenomena very differently; and each states his case very ably. In Mr. Travers' work the views of Hunter on inflammation are fully illustrated, and the various phenomena of the disease delineated with graphic powers of no ordinary description: while in that of Dr. Bennett, a difficult and obscure subject is lucidly treated, and an attempt made to apply to the explanation of anormal productions a theory which has so much contributed to the elucidation of the nature of healthy structure.

